# The Effect of Simplifying Dental Implant Drilling Sequence on Osseointegration: An Experimental Study in Dogs

**DOI:** 10.1155/2013/230310

**Published:** 2013-01-30

**Authors:** Gabriela Giro, Nick Tovar, Charles Marin, Estevam A. Bonfante, Ryo Jimbo, Marcelo Suzuki, Malvin N. Janal, Paulo G. Coelho

**Affiliations:** ^1^Department of Biomaterials and Biomimetics, New York University, 345E 24th Street, Room 813A, New York, NY 10010, USA; ^2^Postgraduate Program in Dentistry, School of Health Sciences, UNIGRANRIO University, Rua Professor José de Souza Herdy, 1.160-25 de Agosto, 25071-202 Duque de Caxias, RJ, Brazil; ^3^Department of Prosthodontics, Faculty of Odontology, Malmö University, Smedjegatan 16, 214 2 Malmö, Sweden; ^4^Department of Operative Dentistry and Prosthodontics, Tufts University School of Dental Medicine, One Kneeland Street, Boston, MA 02111, USA; ^5^Department of Epidemiology and Health Promotion, New York University College of Dentistry, New York, NY 10010, USA; ^6^Department of Periodontology and Implant Dentistry, New York University College of Dentistry, 345E 24th Street, New York, NY 10010, USA

## Abstract

*Objectives*. To test the hypothesis that there would be no differences in osseointegration by reducing the number of drills for site preparation relative to conventional drilling sequence. *Methods*. Seventy-two implants were bilaterally placed in the tibia of 18 beagle dogs and remained for 1, 3, and 5 weeks. Thirty-six implants were 3.75 mm in diameter and the other 36 were 4.2 mm. Half of the implants of each diameter were placed under a simplified technique (pilot drill + final diameter drill) and the other half were placed under conventional drilling where multiple drills of increasing diameter were utilized. After euthanisation, the bone-implant samples were processed and referred to histological analysis. Bone-to-implant contact (BIC) and bone-area-fraction occupancy (BAFO) were assessed. Statistical analyses were performed by GLM ANOVA at 95% level of significance considering implant diameter, time *in vivo,* and drilling procedure as independent variables and BIC and BAFO as the dependent variables. *Results*. Both techniques led to implant integration. No differences in BIC and BAFO were observed between drilling procedures as time elapsed *in vivo*. *Conclusions*. The simplified drilling protocol presented comparable osseointegration outcomes to the conventional protocol, which proved the initial hypothesis.

## 1. Introduction

Osseointegration has been defined as the intimate contact between bone tissue and implanted biomaterial in the optical microscopy level, and such phenomenon has rendered dental implantology as one of the most successful treatment modalities in both dentistry and medicine [1, 2]. However, while high success rates have been reported (often higher than 90% over a decade), the early failure of the osseointegration has been associated with endogenous factors such as quantity and quality of bone, smoking habits, and host systemic impairment, as well as nutritional status and osteometabolic disorders that may impair bone healing or affect the maintenance of osseointegration. On the other hand, especially in cases where endogenous factors are not present, failure of dental implants has also been attributed to exogenous factors such as implant design (including macro- and microgeometry), surgical technique (excessive surgical trauma), overload, misfit of suprastructures, or surgical site infection [3, 4]. 

Albrektsson et al. (1981) suggested that there are 6 factors that determine the success of osseointegration, that is, biocompatibility, design, surface, state of the host bed, surgical technique, and loading conditions [5]. Needless to say, the proposal advocated some 3 decades ago still remains the gold standard for success, and a great number of researches have been conducted on these factors. However, compared to the plethora of studies concerning the implant biocompatibility, design, surface, and loading conditions, the number of studies focusing on the host bed and surgical technique is limited. Especially the effect of surgical procedures such as the drilling protocol has been sparsely explored, and clinicians basically follow the given instructions from the manufacturers. 

Previous research has shown that the osteotomy preparation may result in a region of necrotic bone surrounding the inserted implant and that the extent of this region is potentially influenced by the relationship between the drilling speed and heat generated at these sites [6–8]. Thus, it is expected that the amount of damage incurred to bone due to instrumentation, and subsequently its ability to heal around implants may depend on the drill material, design, whether irrigation is external or internal and if at all utilized, the rate which the drilling site diameter is incrementally increased (the number of iteration from initial drill and final drill diameter prior to implant placement). Different drilling parameters have been currently evaluated in laboratory bench studies, where variations in drilling speed have been shown to be potentially beneficial to implant integration [9, 10]. In addition, heat production during drilling has also been evaluated as a function of drill design [11–14], repeated utilization of drill units [15], and irrigation method [16, 17]. 

With regard to the determination of drilling efficiency and temperature profile as a function of different variables, most investigations are bench studies [9–14, 16, 18], and few represent the osseointegration assessment of implants placed in sites drilled under various conditions [19]. While useful when a numeric control temperature reference is given, these bench studies have not been appropriately validated *in vivo* and such studies are highly desirable.

Even though there are studies investigating the effect of different drilling protocols on osseointegration, little or no data is available regarding the rate in which the drilling site diameter is incrementally increased prior to implant placement, as anecdotally, this procedure has been performed in an incremental drill diameter fashion in an attempt to minimize bone damage during its instrumentation. It is a fact that there is no evidence in the literature whatsoever on the optimal drilling protocol that would result in successful osseointegration in clinical reality. At times, there are drilling protocols that require so many time-consuming steps. It is of great interest to investigate if reducing the number of drills used would provide comparable results to the conventional drilling sequence. Thus, this study tested the hypothesis that no difference in implant osseointegration occurs by reducing the number of drills used for site preparation (pilot drill + final diameter drill) relative to the conventional incremental site preparation.

## 2. Materials and Methods 

This study utilized 72 screw root form endosseous Ti-6Al-4V implants of 3.75 mm (*n* = 36) and 4.2 mm (*n* = 36) in diameter and 10 mm in length (C1, MIS, BarLev Industrial Park, Israel). Half of the implants of each diameter were placed under a simplified technique (pilot drill + final diameter drill) and the other half were placed under the conventional drilling technique where multiple drills of increasing diameter were utilized. Previous atomic force microscopy based texture analysis of the alumina-blasted/acid-etched surface used in the present study were made showing an Sa of 0.35 *μ*m and Sq of 0.5 ± 0.54 *μ*m [20].

Eighteen beagle dogs approximately 1.5 years of age in good health were used in this study under approval of the bioethics committee for animal experimentation at the Ecole Veterinaire D'Alfort, France.

The surgical site was the proximal tibia, a region with a type 2 bone density, and two implants were placed per limb. The right and left limbs provided 3.75 mm and 4.2 mm diameter implants that were placed under the simplified and conventional drilling techniques, respectively (each limb provided samples from the simplified or conventional drilling techniques). 

The conventional drilling sequence for the 3.75 mm diameter implants started from the pilot drill (2.4 mm diameter), an intermediate drill (3.0 mm diameter), and then ended with the final drill (3.6 mm maximum diameter provided with each implant). The conventional drilling sequence for the 4.2 mm diameter implants started from the pilot drill (2.4 mm diameter), two intermediate drills (3.0 mm and 3.5 mm in diameter), and then ended with the final drill (4.0 mm in diameter). The simplified drilling sequence for the 3.75 mm and 4.2 mm diameter implants started with the pilot and then the final burs (3.6 mm and 4.0 mm for the 3.75 mm and 4.2 mm diameter implants, resp.). All drilling procedures were conducted at 900 rpm under abundant irrigation.

## 3. Surgical Procedure

All surgical procedures were performed under general anesthesia. The preanesthetic procedure comprised of an intramuscular administration of atropine sulfate (0.044 mg/kg) and xylazine chlorate (8 mg/kg). General anesthesia was then obtained following an intramuscular injection of ketamine chlorate (15 mg/kg).

Following hair shaving, skin exposure, and antiseptic cleaning with iodine solution at the surgical and surrounding area, a 5 cm incision at the skin level was performed. Then, the flap and muscle layers were reflected and the proximal tibia was exposed.

Two osteotomies were produced at least 10 mm from each other from proximal to distal, and the implants were placed with a torque wrench. Standard layered suture techniques were utilized for wound closure (4–0 Vicryl,internal layers; 4–0 nylon,the skin, Ethicon, Johnson & Johnson, Somerville, NJ). Postsurgical medication included antibiotics (penicillin, 20,000 UI/kg) and analgesics (ketoprofen, 1 mL/5 kg) for a period of 48 h postoperatively.

Euthanasia was performed by an anesthesia overdose (*n* = 6 animals at 1, 3, and 5 weeks after surgery). At necropsy, the limbs were retrieved by sharp dissection, the soft tissue was removed with surgical blades, and initial clinical evaluation was performed. 

## 4. Hard Tissue Histology Preparation

The specimens were fixed in 10% buffered formalin solution for 24 h, washed in tap water for 24 h, and gradually dehydrated in a series of alcohol solutions ranging from 70% to 100% ethanol. Following dehydration, the samples were embedded in a methacrylate-based resin (Technovit 9100, Heraeus Kulzer GmbH, Wehrheim, Germany) according to the manufacturer's instructions. The blocks were then cut aiming at the center of the implant along its long axis with a precision diamond saw (Isomet 2000, Buehler Ltd., Lake Bluff, IL, USA), glued to acrylic slides with an acrylate-based resin, and a 24 h setting time was allowed prior to grinding and polishing. The sections were then reduced to a final thickness of approximately 30 *μ*m by means of a series of SiC abrasive papers (Buehler Ltd., Lake Bluff, IL, USA) in a grinding/polishing machine (Metaserv 3000, Buehler, Lake Bluff, IL, USA) under water irrigation. The sections were then stained in 1% toluidine blue and referred to light microscopy evaluation.

Measurements of the percentages of bone-to-implant contact (BIC) and bone-area-fraction occupancy (BAFO) between threads [21] were performed at 1001x magnification (Leica DM2500M, Leica Microsystems GmbH, Wetzlar, Germany) by using the National Institutes of Health image analyzer software (ImageJ 1.41o, National Institutes of Health, USA). 

The effects of drilling technique, implant diameter, and time *in vivo* on BIC and BAFO were evaluated by a GLM ANOVA. Statistical significance was set at 5% (*α* = 0.05).

## 5. Results

Bone healing around implants was uneventful following implant placement for all 72 sites. No signs of inflammation or infection were observed during the experimental period. 

**Figure 1:**
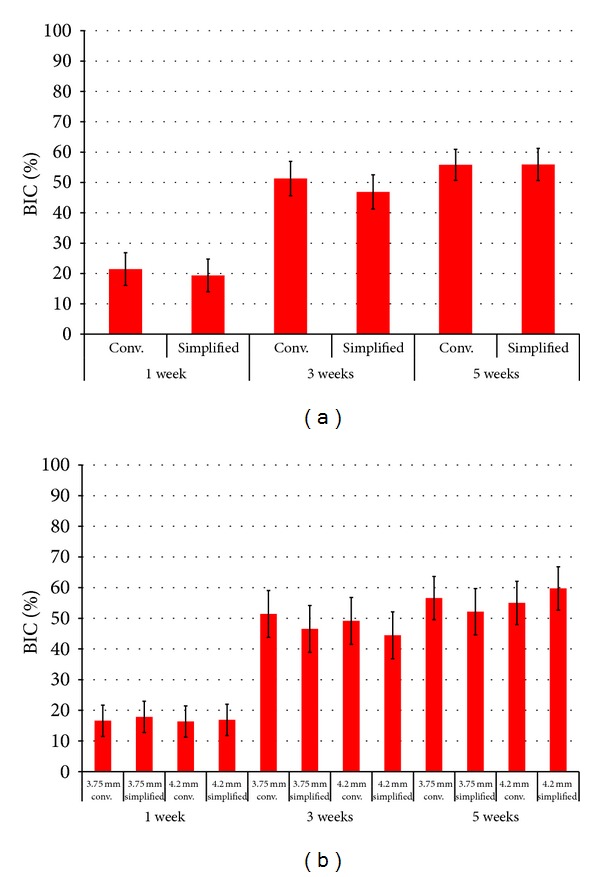
(a) Results for bone-to-implant (BIC) (mean ± 95% CI) as a function of drilling technique and time *in vivo* where no significant differences were observed between groups for each time point *in vivo*. (b) Results for BIC (mean ± 95% CI) as a function of drilling technique, time *in vivo*, and implant diameter. No significant differences were observed between groups for each time point *in vivo*.

The statistical summary concerning the effects of drilling technique as a function of time for BIC is presented in Figure 1(a). While a significant increase was observed from 1 to 3 weeks (*P* = 0.02), this difference was not significant from 3 to 5 weeks (*P* = 0.82). The statistical summary for the effect of drilling technique, implant diameter, and time (Figure 1(b)) did not show significant differences in BIC as a function of drilling technique and implant diameter for each time point evaluated.

**Figure 2:**
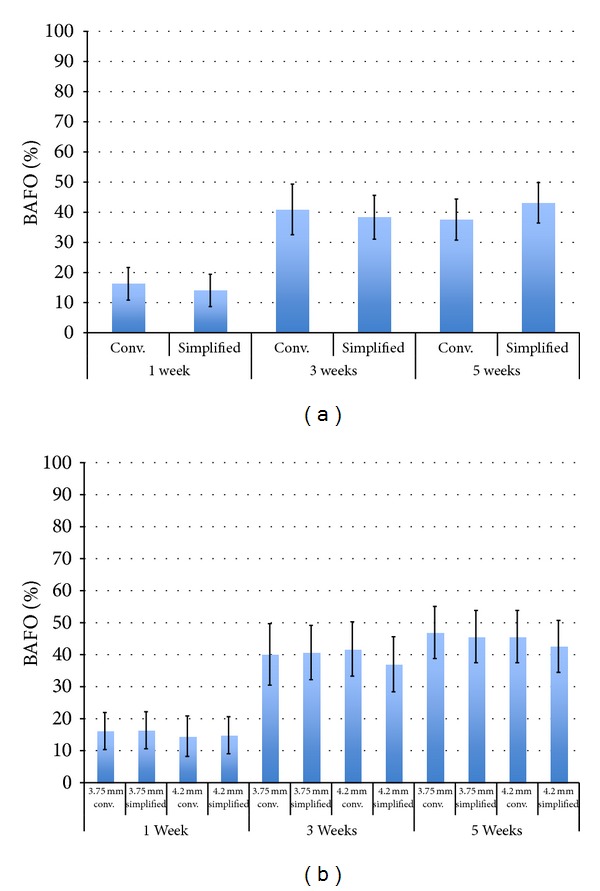
(a) Results for bone area fraction occupancy (BAFO) (mean ± 95% CI) as a function of drilling technique and time *in vivo* where no significant differences were observed between groups for each time point *in vivo*. (b) Results for BAFO (mean ± 95% CI) as a function of drilling technique, time *in vivo*, and implant diameter. No significant differences were observed between groups for each time point *in vivo*.

The statistical summary concerning the effects of drilling technique as a function of time for BAFO is presented in Figure 2(a). While a significant increase in BAFO was observed from 1 to 3 weeks (*P* < 0.01), this difference was not significant from 3 to 5 weeks (*P* = 0.85). The statistical summary concerning the effect of drilling technique, implant diameter, and time (Figure 2(b)) did not depict significant differences in BAFO as a function of drilling technique and implant diameter for each time point evaluated.

**Figure 3:**
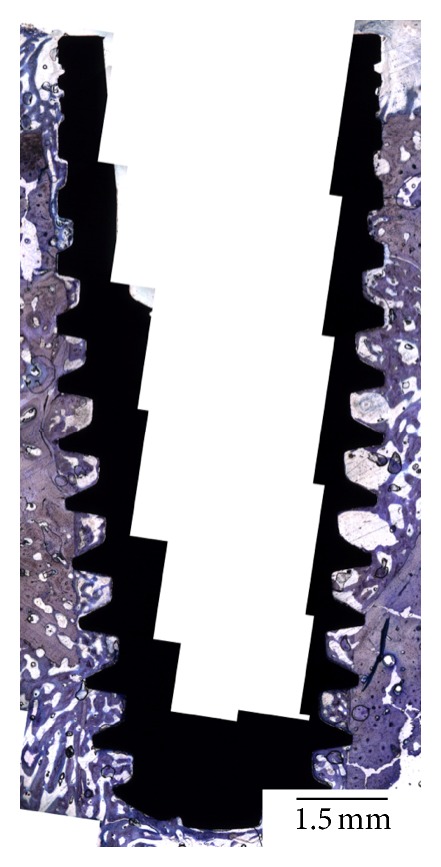
No morphologic differences were observed between implants placed with either conventional or simplified techniques. The evaluation of the histologic sections at all time points showed direct contact between implant and bone in cortical and trabecular regions, as showed in this section of a 4.2 mm diameter implant at 5 weeks of healing.

**Figure 4:**
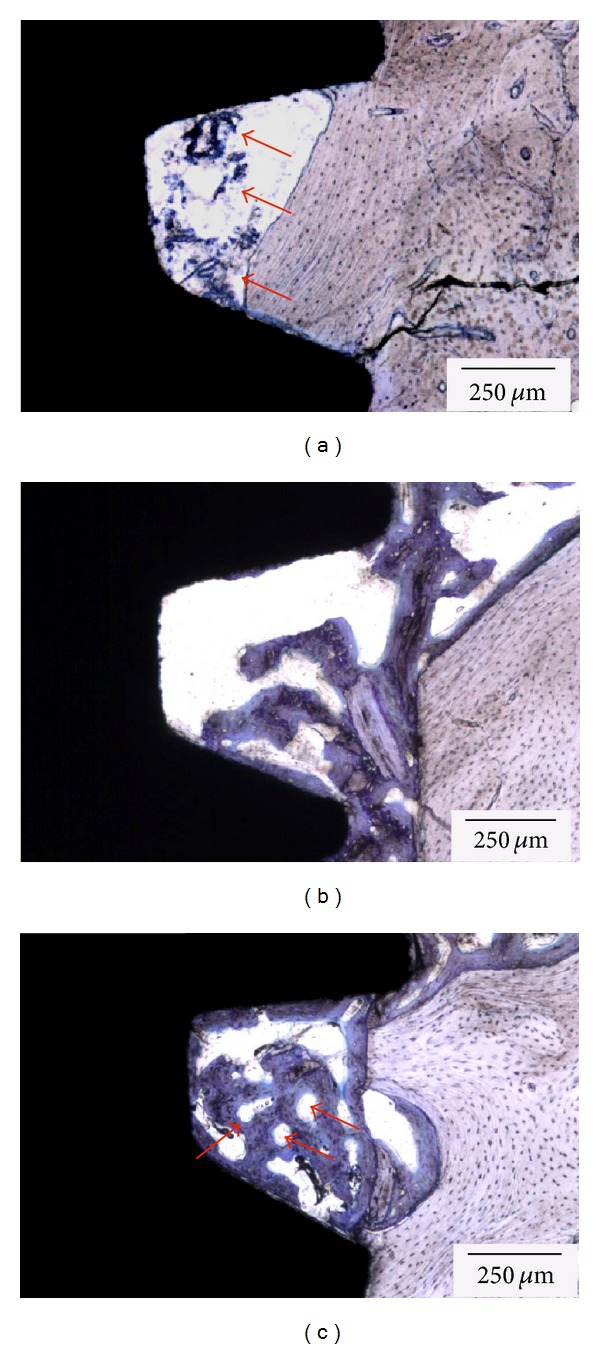
Histologic evaluation showed that at (a) 1 week, initial woven bone formation occurred in the regions between threads and in direct contact with the implant surface (arrows). (b) At three weeks, an increase in the amounts of bone between threads was evident, and the (c) onset of replacement of woven bone by lamellar bone was observed for all groups evaluated at 5 weeks (arrows).

No morphologic differences were observed between implants placed with either conventional or simplified techniques, and initial evaluation of the histologic sections at all time points evaluated showed direct contact between implant and bone in cortical and trabecular regions (Figure 3). In general, the histologic evaluation showed that at 1 week, initial woven bone formation occurred in the regions between threads and in direct contact with the implant surface (Figure 4(a)). At three weeks (Figure 4(b)), an increase in the amounts of bone between threads was evident, and ongoing replacement of woven bone by lamellar bone was observed for all groups evaluated at 5 weeks (Figure 4(c)). 

## 6. Discussion

The present study design allowed the evaluation of osseointegration measurable parameters in implants placed in sites that were prepared in an incremental diameter fashion (conventional) or in a two-step fashion (pilot drill + final drill) to final diameters of 3.6 mm and 4.0 mm at 900 rpm under abundant irrigation. Previous research has pointed that a region of necrotic bone surrounding the implant exists following surgery and that the extent of this region is influenced by drilling speed [9, 10], design [11–14], and irrigation mode (or absence of irrigation) [14, 15]. For most of the research concerning drills and drilling technique variations, the most commonly measured outcome concerns the heat generated at these sites as a function of different variables always referenced by a suitable control group. Thus, while useful when a numeric control temperature reference is given, these studies and the present study hypothesized that no difference in implant osseointegration occurs by reducing the number of drills for site preparation (pilot drill + final diameter drill) relative to the conventional drilling sequence.

It is known that rises in bone temperature during rotary instrumentation are expected to be higher as a function of diametric differences between drills due to the amount of pressure and cutting necessary for site preparation being proportional to this difference. In fact, thermal osteonecrosis is inexorable if the temperature rises higher than 47°C in the bone [22], which has been reported clinically to be one of the causes of implant periapical lesions [23] or otherwise of a delay in bone regenerative process [24]. Intriguingly, not only did our results depict no differences in BIC and BAFO between drilling techniques when implant diameter information was collapsed from statistical analyses, but also showed no difference in BIC and BAFO as a function of implant diameter and time *in vivo*. Further, the histological observation presented no visible differences for both groups, showing no signs of excessive inflammation, osteoclastic activity, or noticeable necrosis. This is an indication that the temperature elevation, if any created by the simplified procedure, did not have any negative effects as compared to the conventional protocol, and the irrigation was probably sufficient enough to keep the temperature below the osteonecrosis threshold of 47°C. If the temperature exceeded 47°C, the healing probably would have delayed for the simplified protocol group, which would have been evident in the histology or in the histomorphometry as reported by Yoshida et al. [24]. Thus, it is highly desirable that future studies combine methods where correlative statistical inferences between temperature rise and osseointegration/biomechanical measurable parameters are possible in order to allow an informed platform for future surgical drilling protocols. 

Since a simplified surgical drilling procedure did not negatively affect the biological response of the implants placed in these sites and was comparable to the conventional drilling sequence, our initial hypothesis that no difference in implant osseointegration occurs by reducing the number of drills for bone site preparation relative to the conventional drilling sequence was accepted. The results of this study strongly suggest that the osteotomy preparation may be simplified and be less time consuming; however, constant irrigation will always be necessary to avoid the deleterious effect of temperature elevation in the bone, specially in high density bone, such as the mandibular anterior region. Lastly, a precise drilling orientation is required in the first drills, as in other techniques, but with fewer opportunities for angulation corrections, which may demand a steeper learning curve for the less experienced professional.

## References

[1] Esposito M, Hirsch JM, Lekholm U, Thomsen P (1998). Biological factors contributing to failures of osseointegrated oral implants. (I). Success criteria and epidemiology. *European Journal of Oral Sciences*.

[2] Brånemark PI, Adell R, Breine U, Hansson BO, Lindström J, Ohlsson A (1969). Intra-osseous anchorage of dental prostheses. I. Experimental studies. *Scandinavian Journal of Plastic and Reconstructive Surgery*.

[3] Albrektsson T, Brunski J, Wennerberg A (2009). ‘A requiem for the periodontal ligament’ revisited. *International Journal of Prosthodontics*.

[4] Esposito M, Hirsch JM, Lekholm U, Thomsen P (1998). Biological factors contributing to failures of osseointegrated oral implants: (II). Etiopathogenesis. *European Journal of Oral Sciences*.

[5] Albrektsson T, Branemark PI, Hansson HA, Lindstrom J (1981). Osseointegrated titanium implants. Requirements for ensuring a long-lasting, direct bone-to-implant anchorage in man. *Acta Orthopaedica Scandinavica*.

[6] Iyer S, Weiss C, Mehta A (1997). Effects of drill speed on heat production and the rate and quality of bone formation in dental implant osteotomies. Part I: relationship between drill speed and heat production. *International Journal of Prosthodontics*.

[7] Augustin G, Davila S, Mihoci K, Udiljak T, Vedrina DS, Antabak A (2008). Thermal osteonecrosis and bone drilling parameters revisited. *Archives of Orthopaedic and Trauma Surgery*.

[8] Reingewirtz Y, Szmukler-Moncler S, Senger B (1997). Influence of different parameters on bone heating and drilling time in implantology. *Clinical Oral Implants Research*.

[9] Sharawy M, Misch CE, Weller N, Tehemar S (2002). Heat generation during implant drilling: the significance of motor speed. *Journal of Oral and Maxillofacial Surgery*.

[10] Kim SJ, Yoo J, Kim YS, Shin SW (2010). Temperature change in pig rib bone during implant site preparation by low-speed drilling. *Journal of Applied Oral Science*.

[11] Chacon GE, Bower DL, Larsen PE, McGlumphy EA, Beck FM (2006). Heat production by 3 implant drill systems after repeated drilling and sterilization. *Journal of Oral and Maxillofacial Surgery*.

[12] Sumer M, Misir AF, Telcioglu NT, Guler AU, Yenisey M (2011). Comparison of heat generation during implant drilling using stainless steel and ceramic drills. *Journal of Oral and Maxillofacial Surgery*.

[13] Oh HJ, Wikesjö UM, Kang HS, Ku Y, Eom TG, Koo KT (2011). Effect of implant drill characteristics on heat generation in osteotomy sites: a pilot study. *Clinical Oral Implants Research*.

[14] Scarano A, Piattelli A, Assenza B (2011). Infrared thermographic evaluation of temperature modifications induced during implant site preparation with cylindrical versus conical drills. *Clinical Implant Dentistry and Related Research*.

[15] Carvalho AC, Queiroz TP, Okamoto R, Margonar R, Garcia IR, Filho OM (2011). Evaluation of bone heating, immediate bone cell viability, and wear of high-resistance drills after the creation of implant osteotomies in rabbit tibias. *International Journal of Oral and Maxillofacial Implants*.

[16] Benington IC, Biagioni PA, Briggs J, Sheridan S, Lamey PJ (2002). Thermal changes observed at implant sites during internal and external irrigation. *Clinical Oral Implants Research*.

[17] Flanagan D (2010). Osteotomy irrigation: is it necessary?. *Implant Dentistry*.

[18] Rashad A, Kaiser A, Prochnow N, Schmitz I, Hoffmann E, Maurer P (2011). Heat production during different ultrasonic and conventional osteotomy preparations for dental implants. *Clinical Oral Implants Research*.

[19] Giro G, Marin C, Granato R (2011). Effect of drilling technique on the early integration of plateau root form endosteal implants: an experimental study in dogs. *Journal of Oral and Maxillofacial Surgery*.

[20] Marin C, Granato R, Suzuki M (2010). Biomechanical and histomorphometric analysis of etched and non-etched resorbable blasting media processed implant surfaces: an experimental study in dogs. *Journal of the Mechanical Behavior of Biomedical Materials*.

[21] Leonard G, Coelho P, Polyzois I, Stassen L, Claffey N (2009). A study of the bone healing kinetics of plateau versus screw root design titanium dental implants. *Clinical Oral Implants Research*.

[22] Augustin G, Davila S, Udilljak T, Staroveski T, Brezak D, Babic S (2012). Temperature changes during cortical bone drilling with a newly designed step drill and an internally cooled drill. *International Orthopaedics*.

[23] Reiser GM, Nevins M (1995). The implant periapical lesion: etiology, prevention, and treatment. *Compendium of Continuing Education in Dentistry*.

[24] Yoshida K, Uoshima K, Oda K, Maeda T (2009). Influence of heat stress to matrix on bone formation. *Clinical Oral Implants Research*.

